# Diagnosis and Management of Fungal Neglected Tropical Diseases In Community Settings—Mycetoma and Sporotrichosis

**DOI:** 10.3390/tropicalmed4020081

**Published:** 2019-05-16

**Authors:** Roberto Estrada-Castañón, Guadalupe Estrada-Chávez, María de Guadalupe Chávez-López

**Affiliations:** 1 Community Dermatology Mexico C.A.; Health Secretary Guerrero, 39355 Acapulco, Guerrero, Mexico; restrada_13@hotmail.com; 2 Department of Dermatology and Dermato-Oncology, Instituto Estatal de Cancerología “Dr. Arturo Beltrán Ortega”, Health Secretary Guerrero, Faculty of Medicine, Universidad Autónoma de Guerrero Mexico, Community Dermatology Mexico C.A., 39850 Acapulco, Guerrero, Mexico; estradaguadalupe@hotmail.com; 3 Department of Dermatology and Mycology Acapulco General Hospital, Health Secretary Guerrero, Community Dermatology Mexico C.A., 39355 Acapulco, Guerrero, Mexico

**Keywords:** subcutaneous mycosis, actinomycetoma, eumycetoma, sporotrichosis Community dermatology

## Abstract

Background: This is a retrospective, analytic observational study where we describe cases of sporotrichosis and mycetoma from Acapulco General Hospital and Community Dermatology Mexico C.A. over 25 years. Analysis of environmental features that favour the development of such diseases has been made, as well as the limitations in the study and treatment of such diseases in resource poor settings. Methods: We reviewed the information on 76 sporotrichosis and 113 mycetoma patients out of a total of 14,000 consultations at Acapulco General Hospital and from Community Dermatology clinics. We analysed the epidemiological and mycological characteristics and the investigations used for diagnosis such as direct examination, culture, intradermal test reactions, and biopsy. Results: In total 91 confirmed cases of actinomycetoma, 22 of eumycetoma and 76 of sporotrichosis have been identified including diagnostic studies for both diseases and their treatment. Discussion: The results obtained have been analysed and interpreted in patients with mycetoma and sporotrichosis in the state of Guerrero, México, along with limitations in their management in areas with limited economic and logistical resources. The prevalence of mycetoma in our setting is compared with other centres where patients from all over the country are seen. The possible causes for variations in prevalence in specific areas has been looked for, in one of the poorest states of the Mexican Republic.

## 1. Introduction

**Figure 1:**
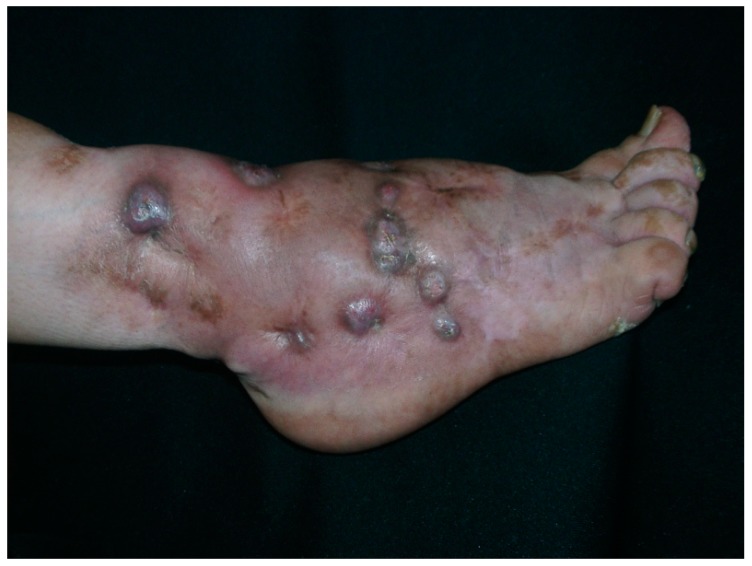
Eumycetoma of the foot.

**Figure 2:**
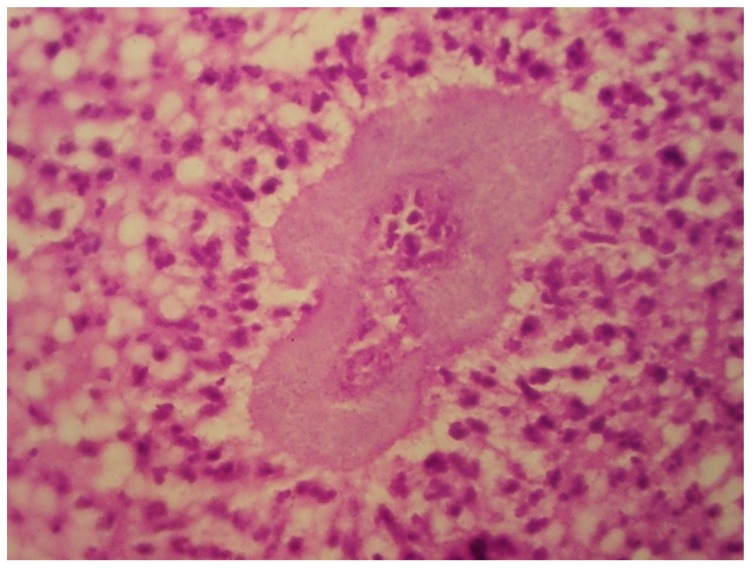
Grain histopathology (actinomycetoma) Haematoxylin and Eosin 100X.

Mycetoma and sporotrichosis are two of the most widely distributed implantation (subcutaneous) mycoses worldwide. Mycetoma is a chronic infectious disease, which can be caused by different species of fungus (eumycetoma) or by aerobic filamentous bacteria (actinomycetoma). Clinical characteristics include, local swelling, and draining sinuses with serosanguinous or purulent exudates (Figure 1), which contains the infective forms known as “grains” (Figure 2) Climatic and geographic conditions have been described previously and the main endemic zone identified as between the Tropic of Cancer at lattitudes 15º south and 30º north; this has been referred to as the “Mycetoma belt” by the World Health Organisation (WHO). It includes countries like Chad, Ethiopia, India, Mauritania, Senegal, Somalia, Sudan, Yemen, and in the Americas: Mexico and Venezuela [1].

Mexico has been identified as the country with the highest incidence of mycetoma in Latin America [2] and one of the most endemic countries around the world [3]. Mycetoma has been recognised by the WHO, as a neglected tropical disease (NTD) in 2016 in a WHA 69.21 resolution [4].

Even though sporotrichosis does not have the same morbidity patterns, except in disseminated or systemic forms, as mycetoma, because of its capacity for dissemination, its severity and neglect; it accounts for significant disability, morbidity, and reduction in the quality of life of affected individuals, who are mostly peasants; these are mainly farmers living in remote areas where diagnosis and treatment is frequently delayed due to lack of experience of local health personal working in the communities. Sporotrichosis is a chronic granulomatous implantation mycosis, caused by a group of dimorphic fungi belonging to the genus, *Sporothrix* . It affects either humans or some animal species [5] It is considered, in some cases, to be an occupational disease, not only in farmers, but also in florists, carpenters, and workers using hay for packing or building. Latin America has reported a high incidence of the infection, particularly in Brazil and Mexico [6], although in the former most cases, in the most recent outbreak, are caused by the zoonotic species *Sporothrix brasiliensis*, spread by cats, unlike the disease in Mexico.

**Figure 3:**
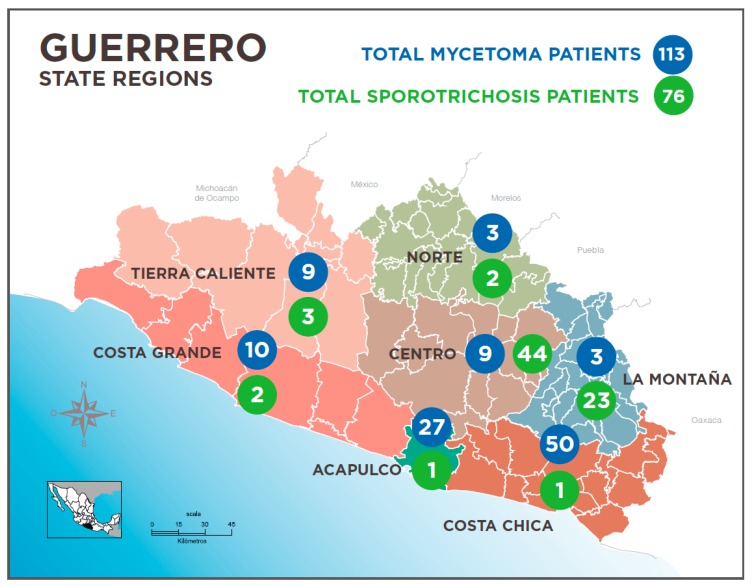
Map of Guerrero State showing cases of mycetoma and sporotrichosis.

Guerrero State in Mexico, is located at the south west of the Mexican Republic, on the coast of the Pacific Ocean between the coordinates 17°36′47″N 99°57′00″O with a territory of 63,794 km^2^; to the West it is crossed by the mountain chain, the Sierra Madre del Sur, which provides very wide climatic and environmental diversity, with valleys and hills suitable for the development of different deep and subcutaneous mycoses. It is divided into seven natural regions, which determined its political division (Figure 3): The North and Mountain areas “Norte” and “Montaña” are arid and with difficult terrain, rich in mining and caves that are frequently visited by cave explorers and tourists, in whom cases of histoplasmosis have been reported [7], the “Tierra Caliente” (warm earth) area, has a wide valley crossed by the Balsas river and very high daily temperatures which provides the name to the region, cases of coccidioidomycosis [8] have been reported from there. A mountain chain divides the central region “Centro” into the other two regions, which are the small and big coastal areas, “Costa chica” and “Costa Grande”, these are rich in vegetation, with coffee and palm plantations, where cases of paracoccidioidomycosis [9] and chromoblastomycosis [10] have been reported. Nevertheless, as in other areas of the world, mycetoma and sporotrichosis are the most frequent subcutaneous mycoses in Mexico [3] and in Guerrero State [11], making it the country with second or third highest prevalence of these disesases [1,2]. 

Mycetoma is more frequent in the Coastal and Tierra Caliente areas. Sporotrichosis is more frequent in the Mountain and Central areas (Figure 3). Affected farmers live in conditions of poverty that can be severe in isolated regions. 

Community Dermatology Mexico (CDM) is a 28 year old programme whose goal is to assist people in need or without access to specialized dermatological care, by teaching basic dermatology to health workers working in remote areas where there is less opportunity for continuous medical education and to identify, refer, and/or treat patients with both simple and complicated dermatological problems and to carry out research [12,13], the reason for this communication. 

## 2. Methods

We report an analytical, retrospective, observational study in which we reviewed 14,000 consultations at the Acapulco General Hospital and records of the Community Dermatology program over the last 20 years. The information obtained in the communities was the result of visits to the areas with a high marginality index amongst the seven regions of Guerrero state, with the support of the State health programme called Desarollo Integral de la Familia or DIF and the Secretary of Health. Of the mycetoma and sporotrichosis cases that were identified clinically, all were referred to our Dermatology Department at the Acapulco General Hospital for further studies and treatment.

Due to limitations associated with field work and the logistics needed for transportation of basic material and equipment, the studies, that were feasible, were restricted to mycological scrapings for direct examination, punch biopsies, and occasionally the inoculation of samples of lesional exudate onto media for subsequent culture, which were subsequently completed in the mycology facility in our institution. All organisms that were speciated in this study were identified by culture. 

In order to facilitate and assist the management of affected patients, we provided financial support for transportation and food during their trip to Acapulco city, where after having any ancillary studies such as imaging including Computer Assisted Tomography (CAT) scans in specific cases, culture, or further mycological studies, free treatment for their specific health conditions was provided after evaluation; this included: Trimethoprim sulfamethoxazole and dapsone (DDS) for patients with actinomycetoma, itraconazole (100–200mg daily), and terbinafine (250 mg daily) for eumycetoma patients and potassium iodide for sporotrichosis; the use of the latter medication is due to cost constraints. 

Despite the support given to the patients, 38% did not attend subsequent consultations because of economic, language, personal reasons or sometimes because family members or friends, raised the spectre of unnecessary amputation. Distance and poverty were constant barriers to consent, study, and treatment.

In order to review cases we developed a basic recording format including, patient information, in Excel and graphics were made with statistics analysis SPSS.9

## 3. Results

Table 1 shows the results from 113 confirmed mycetomas studied. For logistical reasons not all mycological studies could be carried out on all patients. There were more actinomycetomas than eumycetomas with scattered anatomical distributions 

Studies made for confirmation of the diagnosis were: Biopsy in 79 cases, direct examination in 101 and culture in 82 cases. Actinomycetoma cases were caused by *Nocardia brasiliensis* in 64 cases, *Nocardia sp* 19 cases, *Actinomadura madurae* 6, *Nocardia otitidis caviarum* 2. The causative agents in eumycetomas were in most of the cases *Madurella mycetomatis* 16, *Trematosphaeria grisea* 3, *Scedosporium apiospermum* 2, and *Phomopsis longicolla* 1 case. 

Table 2 shows the 76 cases of sporotrichosis studied, of which 43 were of the lymphangitic clinical pattern, fixed type 24 and cutaneous disseminated 8.35 were male, 40 female; 39 adults and 36 children. Most of these patients were farmers; men, women or children work, or are partially involved, in field activities.

The most commonly affected area was the upper limb in 32 cases, 16 in lower limbs, 16 on the face, 4 on the thorax and 3 in other localizations. All cases studied included auxiliary laboratory methods such as culture 52 cases, biopsy in 31 cases, intradermal skin test reaction with mycelial sporotrichin of which 45 cases had positive reactions (one was negative). Of diagnosed cases 56 received treatment with saturated solution of potassium iodide (at full dose 4–6 mls three times daily) with complete healing of the clinical lesions. The rest of the patients did not attend consultation for treatment. Almost 60% of cases occurred in patients in the “Central area” adjacent to the “Mountain area” - for this reason often referred to as the “low mountain” zone—but the other 30% were in the “high Mountain” zone; the remaining 11% were from other areas of the state. 

## 4. Discussion

Neglected diseases in developing countries are usually located in remote, isolated areas with limited access, and communication. This means that health systems are limited, and there is a high level of ignorance and malnutrition all of which are reasons why these diseases develop without intervention and reach extremes of severity, leading to severe morbidity and even death amongst affected patients. One of the main and persistent obstacles to care and further research is the lack of properly trained health personnel to identify patients or to perform studies [14]. Additionally personal safety of staff is a further obstacle to investigation and care, imposing a risk in visits to isolated communities. Even though, encounters with criminal groups have been few and without severe consequences during work in the communities, the potential risk for personnel, has now forced us to adapt our working methods to include the use of teledermatology and telemedicine in order to continue the work of teaching, research, and advice for the health personnel based in remote areas [15].

In 1988, Lavalle and Padilla described in a communication [16] that Guerrero was the second commonest source in the country of mycetoma cases attending the “Centro Dermatológico Pascua” (CDP), which is a referral centre for treatment of dermatological problems. In another review of mycetomas by Bonifaz et al., Guerrero was rated 3rd in incidence (1). In comparing our data with that of Lavalle and Bonifaz for patients coming from Guerrero, but diagnosed and treated in the Department of Mycology at the Hospital General de Mexico (HGM), patient parameters were similar: male predominance in 70%, mostly middle aged, foot involvement, though in our studies uncommon areas such as the perianal region [17] or the neck, a source of considerable morbidity, have also been seen to be involved [18]. Causal agents reported in these other studies are also similar with a predominance of actinomycetes in 97% (Lavalle), 82% (Bonifaz), and 81% (Current study). It is worth mentioning that both centres in Mexico city are among the main primary mycology referral laboratories where patients are seen from every part of the country.

Our studies have also found that there are regional variations in disease prevalence for both mycoses in our environment. Eumycetomas are predominantly found in the Costa Chica area where they account for a much higher proportion of cases (30%) compared with the 2–3% reported nationally. Most sporotrichosis cases originate from the Central and Montaña regions (Figure 3). In explanation, besides the environmental characteristics–in the Costa Chica there is pastureland and cattle farming in commo—it is possible that there is a higher genetic predisposition, related to a high proportion of indigenous Mexican groups, for some mycoses. In the Costa Chica there is also a well established population of individuals of African Mexican origin. It has also been suggested in cases of sporotrichosis, in patients of indigenous origin in the Central Mountain areas carry alleles of Class II [19].

*Madurella mycetomatis* is the most important causal agent found in our series which is similar to studies reported in patients from other areas with high endemicity [20,21,22]; these isolates have not been subject to molecular identification, therefore the presence of other *Madurella* species is possible. Mycetomas per se have a low mortality, except those cases with neck and dorsal spine involvement [18], nevertheless they do have severe morbidity, which has a serious impact on the earning capacity of the affected patients as they are usually farmers with reduced economic resources and depend directly on their ability to work in the fields. The high cost of the treatment and the long term course of the disease, have a critical impact on the family’s health and financial well-being [21].

Histopathological studies are a very valuable diagnostic auxiliary method used to differentiate the actinomycetomas and eumycetomas, for which treatment is completely different. Samples can be easily obtained even in remote areas and together with direct examination and culture can be accessible in areas where resources are extremely limited. Molecular diagnostic techniques are usually out of the reach of decentralized institutions where mycetoma patients are studied and treated [22] and even though simple imaging studies and ultrasound are accessible in order to identify bone involvement, other studies like CAT scan or MRI, can be unaffordable for patients of low income, which represent most of the patients studied in our group [23].

We firmly believe that mycetoma treatment should be as integrated or “holistic” [24]. In our opinion this should include (1) training of the local health personnel, providing the basic knowledge to identify and refer mycetoma cases to centres where they can be properly diagnosed, studied and treated, (2) facilitating the means for patients to attend for consultation, (3) provide appropriate tests for identification of causal agents, together with estimation of the severity index in order to determine the best available treatment for each case, (4) provide free medications throughout treatment duration, and (5) register of every case in order to contribute with information on the epidemiology of mycetoma worldwide (6) establishing a robust system for early detection, and ultimately, prevention of cases in collaboration with local health workers. In the case of sporotrichosis further work needs to be carried out, in order to establish the specific molecular identification of the causative *Sporothrix* species and the reasons for small outbreaks in specific areas, although feline sources of infection are not suspected. As with mycetoma, improving early case recognition is an important goal.

We should point out that even though the number of suspected mycetoma cases diagnosed in Community Dermatology over the last 28 years of work exceeds 240 patients, the diagnosis in many cases has been mainly determined on clinical grounds with some laboratory tests, as discussed previously; in these patients other essential laboratory studies could not be done in order to establish the causal species. Hence, the clinically diagnosed cases have not been included in the analysis.

Finally it is important to mention that 2019 is the year when the Community Dermatology Centre “Dr. Ramon Ruiz Maldonado” (CDM) has been opened in Acapulco in order to treat patients of low and very low incomes from the urban, and especially, rural areas where access to specialized attention is limited or absent.

## Figures and Tables

**Table 1:** Mycetoma Patients.

VARIABLE	VALUES	
GENDER	Male	= 85 (75.2%)
	Female	= 28 (24.8%)
AGE	Adults > 18a	= 104 (92.0%)
	Children < 18a	= 9 (8.0%)
OCCUPATION	Farmers	= 53 (48.6%)
	Housewives	= 23 (21.1%)
	Others	= 37 (30.3%)
AFFECTED AREA	Feet	= 34 (30.1%)
	Legs	= 11 (9.7%)
	Upper limbs	= 22 (19.5%)
	Pelvic area	= 19 (16.8%)
	Abdomen	= 6 (5.3%)
	Trunk	= 17 (15.1%)
	Cervical column	= 4 (3.5%)
MYCOLOGY	Cultures (+)	= 82
	Direct Exam (+)	= 101
	Biopsy	= 79
	X ray	= 75
	CT	= 7
TYPE OF MYCETOMA	Actinomycetoma	= 81 (78.6%)
	Eumycetoma	= 22 (21.4%)
ACTINOMYCETES	*Nocardia brasiliensis*	= 64 (70.4%)
	*Nocardia spp*	= 19 (20.9%)
	*Actinomadura madurae*	= 6 ( 6.6%)
	*N. otitidis caviarum*	= 2 (2.1%)
FUNGI	*Madurella mycetomatis*	= 16 (72.8%)
	*Trematosphaeria grisea*	= 3 (13.7%)
	*Scedosporium boydii*	= 2 ( 9.0%)
	*Phomopsis longicola*	= 1 ( 4.5%)
EVOLUTION	<five years	= 84 (74.3%)
	>five years	= 29 (25.7%)
REGION	Costa Chica	= 50 (44.2%)
	Acapulco	= 27 (23.9%)
	Costa Grande	= 10 (8.8%)
	Tierra Caliente	= 9 (8.0%)
	Centro	= 9 (8.0%)
	Norte	= 5 (4.4%)
	Montaña	= 3 (2.7%)

**Table 2:** Sporotrichosis Patients.

VARIABLE	VALUES	
GENDER	Male	= 35 (46.0%)
	Female	= 41 (54.0%)
AGE	Adults > 18	= 39 (51.3%)
	Children < 18	= 37 (48.7%)
OCCUPATION	Farmers	= 62 (81.6%)
	Students	= 2 (2.6%)
	Children not working	= 12 (15.8%)
AFFECTED AREA	Upper limbs	= 32 (42.1%)
	Lower limbs	= 16 (21.1%)
	Face	= 16 (21.1%)
	Trunk	= 4 (5.3%)
	Other	= 8 (10.4%)
MYCOLOGY	Cultures	= 52 (+)
	IDR (Skin Test) ^1^	= 45 (+)
	Biopsy	= 31
CLINICAL FORM	Lymphangitic	= 43 (56.8%)
	Fixed	= 24 (32.2%)
	Disseminated	= 8 (11.0%)
EVOLUTION	<1 year	= 18 (23.7%)
	<5 years	= 41 (53.9%)
	>5 years	= 17 (22.4%)
REGION	Centre	= 44 (57.9%)
	High Mountain	= 23 (30.3%)
	Others	= 9 (11.8%)
TREATMENT	Treated	= 56 (73.7%)
	Not treated	= 20 (26.3%)

The sporotrichin used was prepared by the Laboratory of Basic Mycology, Universidad Nacional Autonoma de Mexico.
